# Influence of Uterine Displacements on the Sterile Condition

**Published:** 1865-11

**Authors:** 


**Influence of Uterine Displacements on the Sterile Condition.**

Dr. J. Marion Sims, at the late meeting of the British Medical Association, said that we were all interested in the subject of sterility, when we remember the fact that every eighth marriage was sterile. He did not propose then to give us a complete paper on the subject, but only to present it in one of its relations, viz., that of its dependence upon misplacements of the uterus. He divided his sterile patients into two classes: 1st. Those who were married a sufficient length of time and did not conceive; 2d. Those who had borne children, but for some reason ceased to do so long before the termination of the child-bearing period. The first he called "natural sterility." the second, "acquired sterility."

To show the frequency of uterine displacements in this relation, he said that of 250 cases of "natural sterility" that had fallen under his observation, 103 had anteversion, and 68 retroversion; and of 255 cases of "acquired sterility, 61 had anterversion, and 111 retroversion, the anteversions predominating in the first class, the retroversions in the second, the two opposite displacements being almost in inverse proportion in the two classes and forming about two thirds of the whole number, being 343 out of 505 cases; which proved beyond question the bearing and importance of these displacements in connection with the sterile condition. He then illustrated by diagrams the normal position and relations of the uterus, explained the various causes and complications of anteversion, whether dependant upon fibroid tumors, elongation of the infra or supra-vaginal cervix, shortening of the utero-sacral ligaments, or hypertrophy of the fundus. In all these cases, he said, we could not do much for the relief of the sterile condition by merely mechanical means; that our efforts should be directed to seeing that the os tincæ was properly open, that the canal of the cervix was free from engorgement, and that the secretions, both vaginal and cervical, were not poisonous to the spermatzoa. He said that there was one form of anteversion that was easily cured by a simple and novel operation, which he originated some eight or nine years ago. He illustrated this by cases and diagrams. It was as follows: The uterus lies down on the anterior wall of the vagina, and parallel with it. The fundus is most usually the seat of a fibroid growing anteriorly. The anterior wall of the vagina is greatly elongated, the os tincæ pointing directly backwards. Under these circumstances he has shortened the anterior wall of the vagina an inch and a half, by denuding the surface a half inch wide and two inches long across the axis of the vagina in juxtaposition with the cervix uteri, and making a similar transverse scarification parallel with the first, about an inch and a half, more or less anteriorly to it and then united these two transverse cut surfaces by silver sutures, just as we would unite the edges of a transverse vesico-vaginal fistula by them. This necessarily shortens the elongated auterior wall of the vagina, draws the cervix forwards into its normal relations, and as a consequence elevates the fundus. He related several successful cases of this operation, and had seen it followed by conception and child-bearing. He then passed to the consideration of retrovertion as influencing the sterile condition, pointed out its varieties and anomalies, and showed how it was to be diagnosed and how replaced. By diagrams, he illustrated various modes of reduction, showed how conception was difficult, and sometimes impossible, in some forms of retroversion, advocated mechanical treatment, pointed out the danger of pessaries, but advocated their use when judiciously applied under proper circumstances. He prefers a malleable ring, either of block tin or a ring of copper wire covered with gutta percha, and then bent or curved to the proper diameters of the vagina of each patient. He said this was a modification of Hodge's pessary. Under some circumstances he also uses Meigs's ring pessary, made of watch-spring covered with gutta percha. He pointed out the peculiar advantages of each of these, and paid a just tribute to his countrymen, Drs. Hodge and Meigs, who were the earliest advocates of the mechanical treatment of uterine displacements. He said that the great secret of treating the sterile condition when dependent upon retroversion was to adjust a malleable ring which would hold the uterus in its normal position, and which was to be worn always during the act of coition. He explained its philosophy, its safety, and its harmlessness, and related a great many cases in which its use has been followed by conception: one after a sterile marriage of six years, another of ten years, another of fifteen years, and others at various periods of time after sterile marriages. He also showed how miscarriages, often dependent upon this displacement, are prevented by the use of a properly fitted malleable pessary. He then pointed out the course to be adopted when it was impossible for the patient to wear a pessary, showing why it was so, and what was to be done.—*Med. Times and Gazette.*, Aug. 19, 1865.—*Cincinnati Lancet & Observer.*

